# Development of Cosmetic Emulsions Enriched with *Pinus nigra* Extracts Prepared by Different Extraction Approaches: Physicochemical Stability, Biological Activity and Taxifolin Permeability

**DOI:** 10.3390/ijms27198877

**Published:** 2026-10-06

**Authors:** Nikolaos Bikiaris, Magdalena Paczkowska-Walendowska, Judyta Cielecka-Piontek, Nikolaos Nikolaidis

**Affiliations:** 1 Laboratory of Chemistry and Technology of Polymers and Dyes, Department of Chemistry, Aristotle University of Thessaloniki, 54124 Thessaloniki, Greece; nikompik@pharm.auth.gr (N.B.); nfnikola@chem.auth.gr (N.N.); 2 Department of Pharmacognosy and Biomaterials, Poznan University of Medical Sciences, Rokietnicka 3, 60-806 Poznan, Poland; jpiontek@ump.edu.pl

**Keywords:** emulsions, oil in water (O/W), *Pinus nigra* Arnold, black pine, plant extracts, antioxidant properties, sustainable cosmetics

## Abstract

The growing demand for natural cosmetic ingredients has increased interest in plant-derived extracts with multifunctional biological properties. In this study, oil-in-water (O/W) cosmetic emulsions containing *Pinus nigra* needle extracts obtained by different extraction methods (Soxhlet, reflux, hot oil maceration, and cold oil maceration) and a commercially available essential oil were developed and comprehensively characterized. The formulations were evaluated for physicochemical stability (pH, viscosity, and color), total phenolic content (TPC), taxifolin content, antioxidant, anti-inflammatory, and anti-tyrosinase activities, as well as taxifolin permeability using the Skin-PAMPA model. Water-based extracts, particularly the reflux extract, exhibited the highest TPC (8.02 ± 0.63 mg GAE/mL cream), while formulations containing water extracts demonstrated the greatest antioxidant activity (79.72 ± 5.38% (reflux) and 76.78 ± 4.36% (soxhlet) DPPH inhibition). In contrast, oil-based extracts showed higher anti-inflammatory activity, with hyaluronidase inhibition reaching 85.25 ± 0.89%, and enhanced taxifolin permeability (50.56 ± 2.82 × 10^−6^ cm/s). Principal component analysis indicated a positive correlation between TPC and antioxidant activity (r = 0.806), whereas permeability was negatively correlated with TPC and antioxidant activity but positively associated with oil-based formulations. All emulsions exhibited satisfactory physicochemical stability during storage. Different extraction approaches were associated with distinct chemical and functional profiles of the resulting *P. nigra*-based cosmetic emulsions.

## 1. Introduction

Driven by the global shift toward green and sustainable practices, the utilization of natural ingredients in personal care products has increased extensively in recent decades [1]. Plant-based biochemicals exhibit several desirable characteristics, including environmental compatibility, low toxicity, and inherent sustainability [2]. The cosmetic industry is among the key sectors exploiting phytochemicals for their well-documented bioactive and health-promoting properties. Cosmetic products, such as creams, lotions, lipsticks, etc., are applied directly to external body parts mainly for beautification purposes. However, the use of synthetic chemicals in cosmetics may expose the human skin system to various harmful chemicals and toxic heavy metals. Therefore, the replacement of synthetic chemicals with plant-based natural ingredients helps to obtain safe, ecofriendly, and sustainable cosmetic products [3]. Plant species are a valuable source of biologically active compounds that might be exploited in the cosmetic and pharmaceutical industry [4]. Emphasis is given to the use of black pine tree extracts rich in phenolics for improving oxidative stability of cosmetic emulsions.

Natural products remain one of the most enduring and productive sources of structurally diverse bioactive molecules in pharmaceutical, nutraceutical, and cosmeceutical development. There is increasing demand for cosmetic emulsions with natural active components such as plant extracts because of their numerous benefits. The quality of cosmetic emulsions may be affected by distribution and storage processes, which can lead to peroxidation of lipid components. Lipid peroxidation results in undesirable alterations in the efficacy, texture, and appearance of the cosmetic product, thus indicating a need to find a safe and potent compound to be added to products to postpone oxidation processes.

Emulsions are thermodynamically unstable yet kinetically stable mixtures of two or more immiscible liquids, typically an aqueous and an oil phase, whose stability is achieved through the incorporation of suitable emulsifiers [5]. Depending on the ratio and nature of its phases, an emulsion may be classified as an oil-in-water (O/W) or water-in-oil (W/O) system, each offering distinct sensory and functional properties [6]. Owing to their versatility, emulsions represent one of the most widely used delivery systems in the cosmetic industry, providing an effective vehicle for the incorporation and controlled release of bioactive ingredients [7].

Pines are among the most important and widespread forest trees, as they occur in a different range of environmental and climate conditions. *Pinus nigra* Arnold, or black pine, is one of the most important European conifer species because of its outstanding properties. It is one of the tree species most often introduced outside its native distribution area and planted in the most diverse habitats, from xerothermic locations to mesophilic forests to floodplain forests [8]. Black pine (BP, *Pinus nigra*) needles are a source of various bioactive compounds, particularly phenolic constituents, including flavonoids and phenolic acids, as well as terpenoid compounds. Among the flavonoids, taxifolin is of particular interest due to its antioxidant and other biological properties. The presence of these phytochemicals may contribute to the antioxidant and enzyme-inhibitory activities of black pine extracts, making them potentially valuable ingredients for cosmetic formulations aimed at protecting the skin against oxidative stress and supporting the maintenance of skin structure and appearance [9,10]. Essential oils isolated from different pine species are widely used as fragrances in the cosmetic industry as well as active ingredients in skincare formulations due to their rich monoterpene profile. These volatile oils provide antimicrobial, antioxidant, and anti-inflammatory properties that protect products from contamination and soothe the skin. Additionally, they enhance the transdermal delivery of other active components in lotions, soaps, and hair care products [11,12].

The application of plant extracts in cosmetics has been widely reported in the literature. Grzeszczak et al. [13] investigated the antioxidant capacity of α-Pinene-enriched emulsions isolated from *Pinus sylvestris* L., *Rosmarinus officinalis*, *Levisticum officinale*, and *Salvia officinalis.* Zosimidou et al. [14] prepared emulsions containing *hippophae* oil isolated via different extraction methods and studied their antioxidant, sun-blocking, and physicochemical properties. The group of Adeel evaluated the antioxidant effects and the sun protection factor (SPF) of apple (*Malus domestica*), banana (*Musa sapientum*), and orange (*Citrus reticulata*) peel extracts in skincare formulations [3].

To the best of our knowledge, this is the first study to comparatively evaluate O/W cosmetic emulsions incorporating laboratory-prepared *P. nigra* needle extracts obtained using aqueous and oil-based extraction approaches, alongside a formulation containing commercially available *P. nigra* essential oil as an external comparator. Soxhlet and reflux extraction were both included as conventional hot aqueous extraction approaches to examine whether differences in the mode of solvent–matrix contact and thermal history could translate into differences in the chemical characteristics and functional performance of the resulting formulations. Although both procedures employed water as the extraction solvent, they represent distinct extraction configurations and may differ in extraction dynamics and in the exposure of phytochemicals to thermal conditions. By maintaining a comparable base formulation, the study evaluates whether different extraction approaches are associated with differences in the measured chemical characteristics and functional properties of the resulting emulsions.

## 2. Results

The current study evaluated the properties of various cream formulations enriched with BP extracts by analyzing their active components, biological activities, and permeability characteristics. The creams were assessed in terms of pH and viscosity stability, color, antioxidant, anti-inflammatory, and anti-tyrosinase activities, which are critical indicators of their therapeutic and cosmetic potential. The results provide a comparative characterization of formulations containing *P. nigra*-derived ingredients prepared and incorporated using different aqueous- and oil-based approaches.

### 2.1. Content of Active Components

The total phenolic content and taxifolin levels were measured across the formulations, and the summary of the results is presented in Table 1. Cream 3 exhibited the highest TPC (8.02 ± 0.63 mg GAE/mL), significantly surpassing other formulations. In contrast, Cream 5 displayed the lowest TPC (2.32 ± 0.24 mg GAE/mL), indicating minimal phenolic contribution.

In terms of taxifolin content, Creams 1 and 5 showed the highest levels (1.84 ± 0.01 mg/g and 1.83 ± 0.01 mg/g, respectively), highlighting their potential for delivering this bioactive compound. Conversely, Cream 4 exhibited the lowest taxifolin content (1.59 ± 0.01 mg/g).

### 2.2. Physicochemical Evaluation of Emulsions

#### 2.2.1. pH and Viscosity Stability During Storage

**Figure 1:**
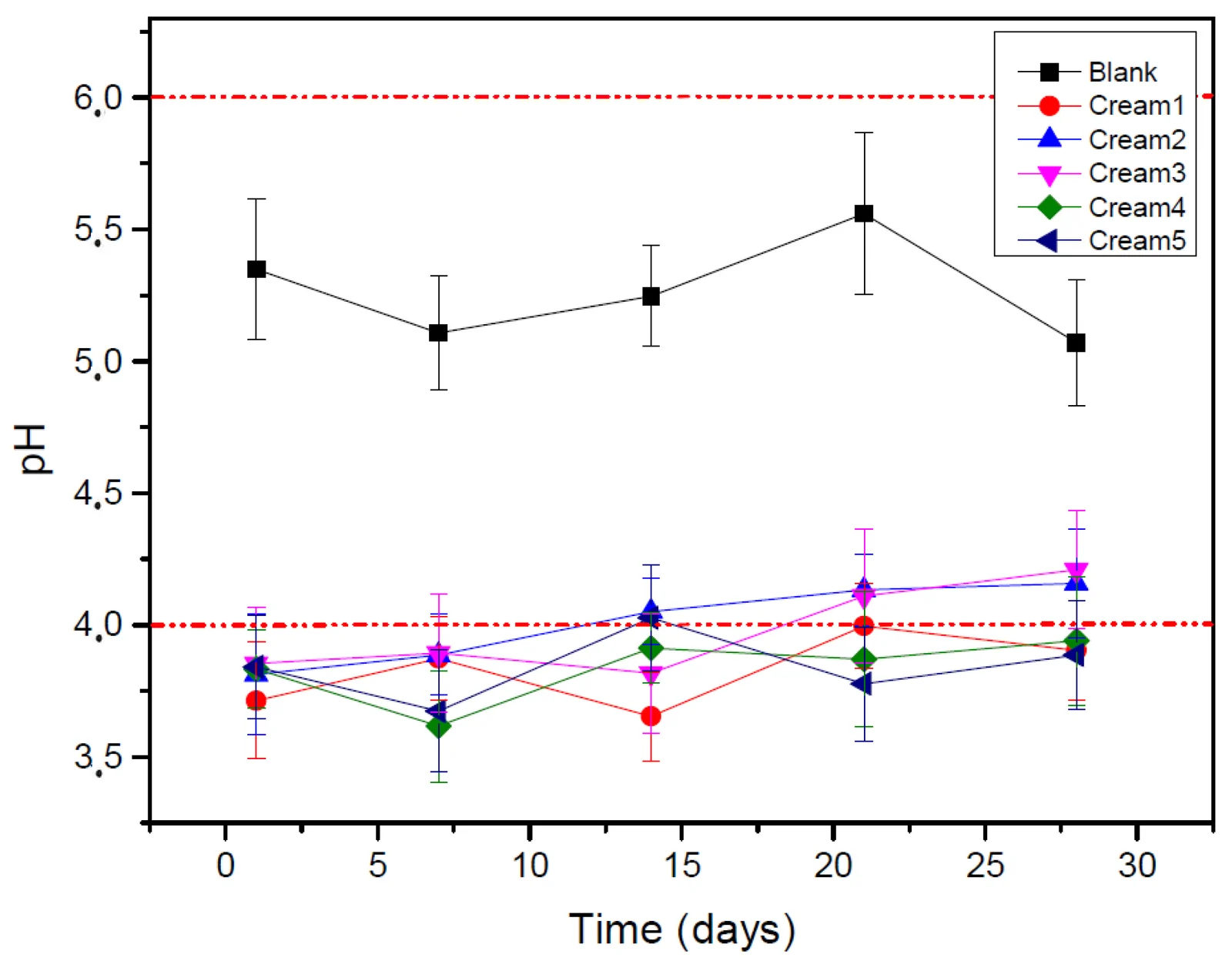
Change in pH values over 28 days of storage at room temperature for the blank emulsion and the BP-enriched O/W emulsions. BP, black pine (*Pinus nigra*); O/W, oil-in-water. Cream 1, BP essential oil; Cream 2, Soxhlet aqueous extract; Cream 3, reflux aqueous extract; Cream 4, hot olive-oil macerate; Cream 5, cold olive-oil macerate. Data are presented as mean ± standard deviation (n = 3). Dashed lines indicate a pH range between 4.0 and 6.0.

The stability of the studied O/W emulsions was evaluated by monitoring their pH and viscosity during storage over a period of 28 days. Throughout this period, the BP emulsions maintained highly acidic pH values, in some cases lower than 4, which do not fall within the acceptable range for dermal cosmetic applications (Figure 1). Interestingly, creams 2 and 3 formulated with DI as the extracting solvent exhibited pH values above 4 at Days 21 and 28, suggesting a convergence toward more neutral/basic conditions during storage. This pH drift could be attributed to the slow degradation, oxidation, or neutralization of acidic constituents in emulsions containing plant extracts, as well as the limited buffering capacity of the vehicle, and can lead to a progressive increase in pH over time. Regarding the blank emulsion, pH values remained between 5.0 and 5.6 throughout storage, i.e., mildly acidic and within the physiological range of the skin surface rather than in the alkaline region. This behavior is attributed to its composition (Table 1), which contains no buffering agent: the only water-soluble ionizable components of the aqueous phase are disodium EDTA, whose solutions are mildly acidic and which exerts only a weak buffering effect, and xanthan gum, supplied as its sodium salt and giving essentially neutral dispersions. The principal role of disodium EDTA is the sequestration of trace transition-metal ions, which suppresses metal-catalyzed oxidation and hydrolysis of the lipid phase and thereby limits the accumulation of free fatty acids and other acidic degradation products during storage [15]. Repeated measures ANOVA analysis did not reveal an effect of time (*p* = 0.106) within the group of formulations. Blank emulsion without the addition of BP extracts yielded significantly higher pH values (*p* = 1) than the BP-containing creams. In addition, using water instead of oil as the solvent did not result in any significant differences in pH values. Post hoc pairwise comparisons with Tukey’s correction revealed no significant differences in emulsion pH between any of the sampling days (*p* > 0.05), indicating that the formulations were pH-stable over 28 days [16].

**Figure 2:**
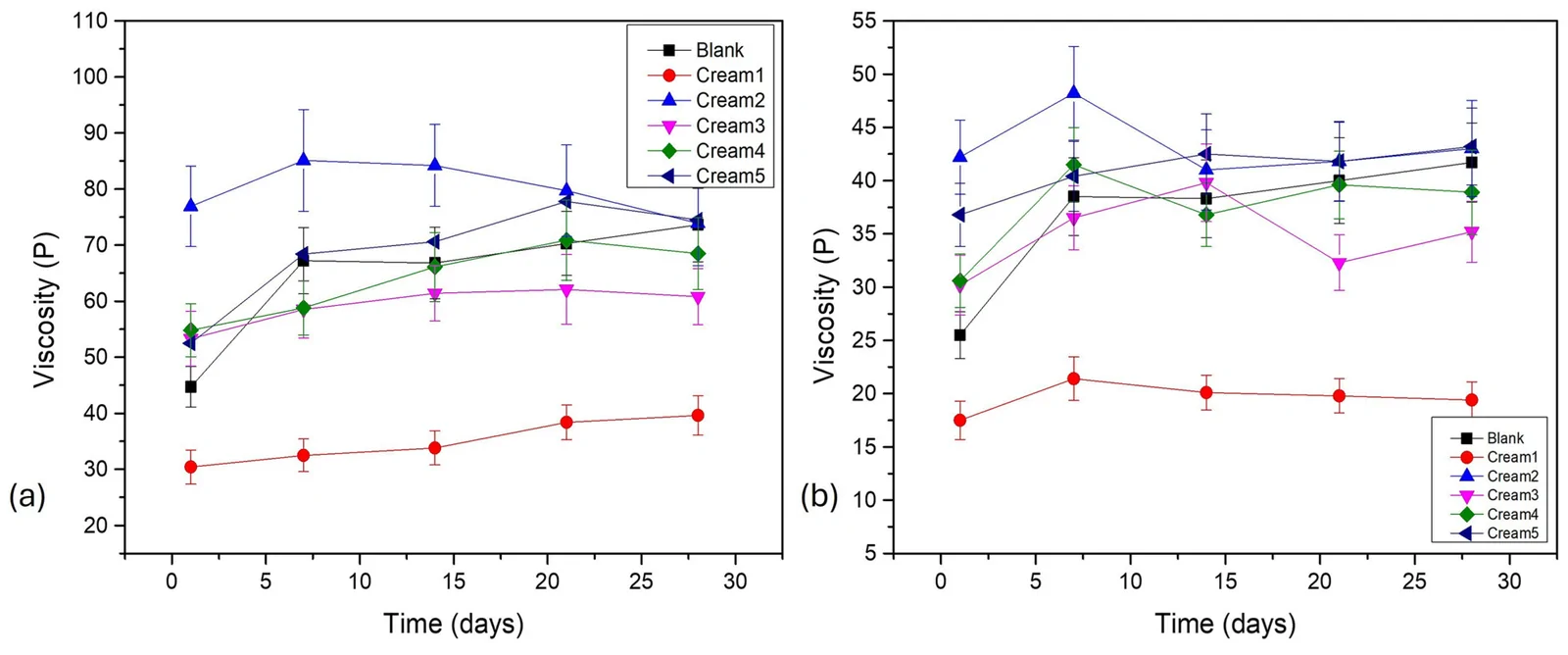
Apparent viscosity of the BP-enriched O/W emulsions during 28 days of storage at room temperature, measured with an R3 spindle at (**a**) 50 rpm and (**b**) 100 rpm, expressed in poise (P). Data are presented as mean ± standard deviation (n = 3).

The flow characteristics of the BP emulsions were assessed throughout a 28-day timeframe, with measurements conducted at rotational speeds of 50 and 100 rpm (Figure 2a,b). These results provide valuable information regarding the response of the emulsion to shear stress. A one-way analysis of variance (ANOVA) demonstrated a statistically significant effect of emulsion formulation on viscosity (*p* < 0.001). Furthermore, repeated measures analysis revealed that temporal variation significantly influenced viscosity values at both shear rates examined (50 rpm and 100 rpm, *p* < 0.001 for both conditions), indicating progressive changes in the rheological properties of the emulsions over the observation period. At 50 and 100 rpm, Cream 1 was found to exhibit the lowest viscosity values on all days, significantly differing from the rest of the emulsions. The significantly lower viscosity exhibited by Cream 1 at both shear rates throughout the study period suggests that the source and composition of the essential oil exert a considerable influence on the rheological properties of the emulsion. The commercial essential oil derived from black pine (Cream 1) demonstrated reduced viscosity compared to laboratory-prepared extracts, which may reflect differences in chemical composition, molecular weight distribution, or the concentration of key bioactive compounds. At 50 rpm, Cream 2 demonstrated statistically significant increases in viscosity relative to the remaining formulations at both the 7-day (85.1 ± 9.1 P) and 14-day (84.2 ± 7.3 P) assessment intervals, indicating better structural integrity during the critical early stabilization period, which could be favorable for dermatological applications. Across all formulations, viscosity measurements obtained at the higher shear rate (100 rpm) were reduced relative to those recorded at the lower shear rate (50 rpm). This finding is characteristic of shear-thinning or pseudoplastic flow behavior, commonly documented in cosmetic emulsion systems. The decrease in viscosity with increasing shear rate indicates that these emulsions behave as non-Newtonian fluids, meaning they flow more easily as greater shear stress is applied.

#### 2.2.2. Color Measurement

The colorimetric analysis of the creams revealed a significant overall effect on a*, b*, and C (Chroma) parameters (L*: *p* = 0.202; a*: *p* < 0.001; b*: *p* < 0.001; C: *p* < 0.001, Table 2). Regarding lightness, no significant differences were observed among the prepared emulsions, indicating that the incorporation of BP extracts does not markedly influence this parameter. This suggests that the visual appearance of the formulations, in terms of perceived brightness, remains largely unaffected by the type or presence of BP extract within the concentration range studied. Creams 4 (−1.093 ± 0.041) and 5 (−0.867 ± 0.042) exhibited significantly lower red–green (a*) values than the other formulations, suggesting that the use of oil as the solvent influences this color parameter. Creams 2 (7.036 ± 0.232) and 3 (6.73 ± 0.33) exhibited the highest chroma values, indicating that the use of deionized water results in emulsions with more vivid color intensity, a characteristic often associated with higher consumer appeal in cosmetic products.

### 2.3. Biological Activities of the Emulsions

Antioxidant activity was evaluated based on the inhibition of DPPH radicals (Table 3). Creams 3 and 2 exhibited the highest antioxidant activities (79.72 ± 5.38% and 76.78 ± 4.36%, respectively), significantly differing from the rest of the emulsions. These results align with their high TPC values (Table 1), reaffirming the correlation between phenolic content and antioxidant potential. In contrast, Creams 1, 4, and 5 demonstrated low antioxidant activities, with values close to 10%, indicating their limited efficacy in free radical scavenging.

The anti-inflammatory potential was measured through hyaluronidase inhibition. Cream 4 showed exceptional performance, with the highest inhibition rate (85.25 ± 0.89%), suggesting its strong ability to mitigate inflammation. The reference cream exhibited the lowest anti-inflammatory activity (11.29 ± 1.20%).

Cream 1 achieved the highest tyrosinase inhibition (51.84 ± 2.49%), indicating its suitability for depigmentation and cosmetic applications. In contrast, the reference cream had the lowest tyrosinase inhibition (9.37 ± 0.81%), suggesting minimal impact on reducing pigmentation.

### 2.4. Permeability of Taxifolin

For topical cosmetic formulations, the ability of bioactive compounds to permeate the skin is an important factor influencing their potential local availability and biological effects. In this context, taxifolin was selected as a representative phenolic compound of *Pinus nigra* extracts to evaluate whether the composition of the cream formulation may affect its permeability. The Skin-PAMPA model provides a useful in vitro screening approach for assessing passive permeation across a membrane mimicking the barrier properties of the skin and allows the comparison of permeability, evaluated using the apparent permeability coefficient (P_app_), between different formulations.

Cream 4 demonstrated the highest permeability (50.56 ± 2.82 × 10^−6^ cm/s), followed closely by Cream 5 (45.65 ± 2.20 × 10^−6^ cm/s), as presented in Table 4. Creams 4 and 5 showed higher taxifolin permeability than the formulations containing aqueous extracts. However, the present experimental design does not allow these differences to be attributed specifically to the oil matrix, as other compositional factors may also contribute. A possible mechanism may involve interactions between lipophilic components of the oil matrix and the membrane, potentially modifying taxifolin partitioning and facilitating its passive diffusion. This interpretation remains hypothetical and requires confirmation in further mechanistic and ex vivo permeation studies. In contrast, Cream 3 exhibited the lowest permeability (26.99 ± 2.00 × 10^−6^ cm/s), suggesting that its high phenolic and taxifolin content did not translate to enhanced delivery.

### 2.5. PCA Analysis

**Figure 3:**
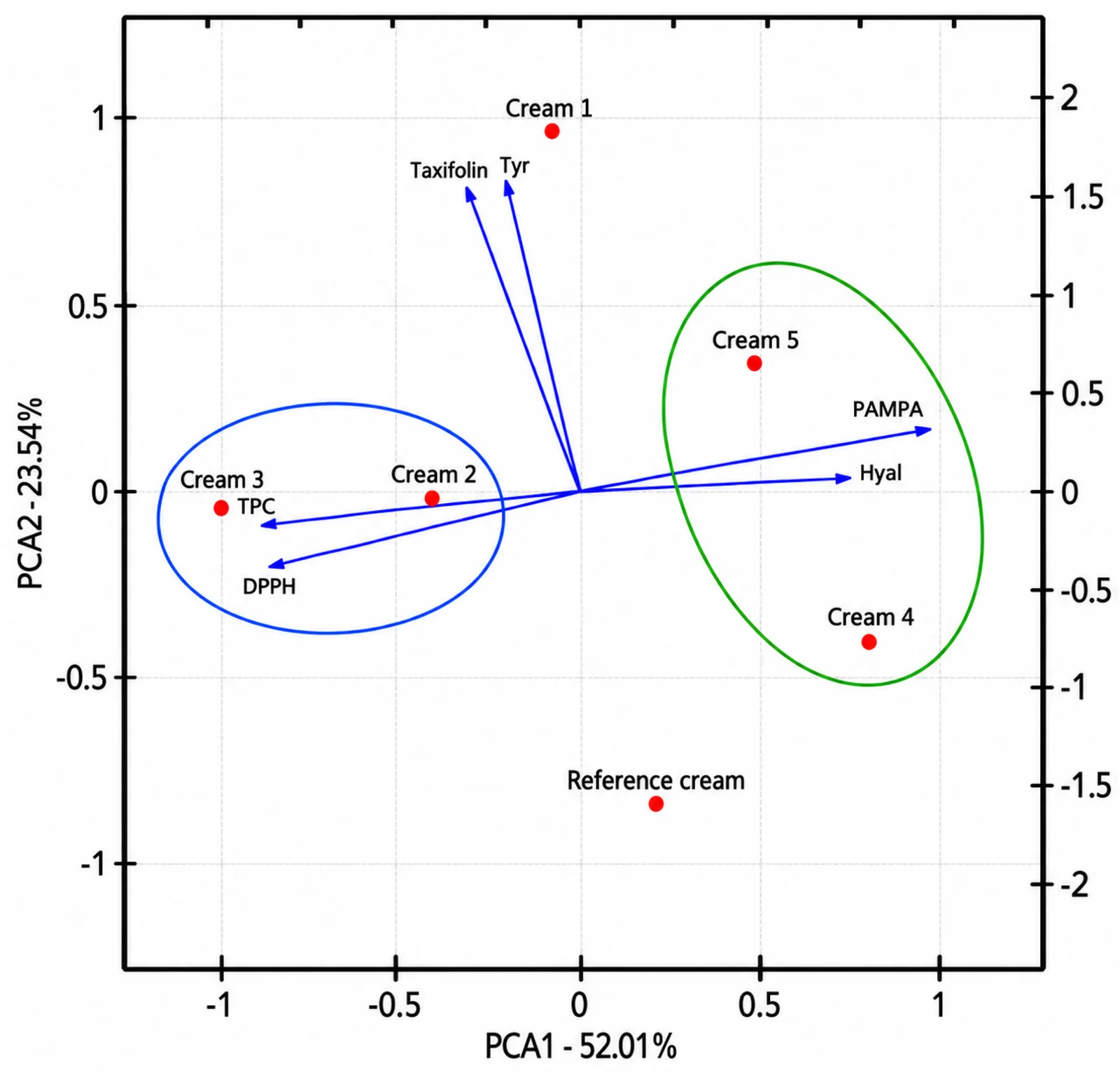
Principal component analysis (PCA) biplot of the characteristics of the BP-enriched emulsions. PC1 and PC2, first and second principal components, with the percentage of total variance explained indicated on each axis.

Principal component analysis (PCA) was used as an exploratory tool to visualize relationships among the investigated formulations and variables. In addition, correlation coefficients were examined to describe the direction and strength of associations between selected parameters (Figure 3). PC1 was mainly associated with antioxidant-related variables, including total phenolic content (TPC) and DPPH radical scavenging activity, whereas PC2 was primarily influenced by taxifolin permeability and anti-inflammatory activity. Antioxidant activity is strongly positively correlated with TPC (r = 0.806), indicating that higher TPC was associated with higher antioxidant activity. Similarly, tyrosinase enzyme inhibition activity is positively associated with taxifolin content (r = 0.478), indicating a moderate positive association, although other constituents of the extracts may also contribute to the observed anti-tyrosinase activity. Taxifolin permeability, however, is weakly negatively correlated with taxifolin content (r = −0.177) and strongly negatively correlated with TPC (r = −0.782) and antioxidant activity (r = −0.811), highlighting a potential limitation in formulations with higher concentrations of these components. Although taxifolin contributes to TPC, TPC represents the overall pool of phenolic compounds. Taxifolin levels varied only slightly among the formulations, while TPC showed considerably greater differences. This suggests that other phenolic constituents may contribute to the observed variation in TPC; however, their identity and relative contribution cannot be established without comprehensive phytochemical characterization of the intermediate extracts. Formulations containing oil macerates were associated with higher taxifolin permeability; however, this association does not establish a causal effect of the oil matrix. Furthermore, formulations containing oil macerates were also associated with higher hyaluronidase inhibition.

The analysis also identifies two distinct groups among the formulations. Creams 2 and 3 contained aqueous extracts, whereas Creams 4 and 5 contained olive-oil macerates, reflecting the impact of the base composition on their physicochemical and biological properties. These findings provide valuable insights into how formulation components influence biological activities, offering a pathway for optimizing product development.

## 3. Discussion

Natural ingredients have been extensively utilized in dermatological and therapeutic applications for centuries. In recent years, there has been renewed interest in plant-derived compounds due to their perceived safety and suitability for incorporation into pharmaceutical and cosmetic formulations. The inclusion of plant extracts in cosmetic emulsions is particularly promising, as their notable antioxidant activity can contribute to inhibiting lipid peroxidation and thereby enhance formulation stability during storage [17]. Plant extracts are widely incorporated into topical formulations for both skin care and the management of dermatological conditions, owing to their favorable safety and biocompatibility. Among these, *Pinus nigra* extracts have attracted considerable interest due to their richness in phenolic compounds, including flavonoids and procyanidins. These bioactive constituents exhibit pronounced antioxidant activity, along with anti-inflammatory and antimicrobial effects, which are relevant for mitigating oxidative stress–induced skin damage and supporting skin barrier function. Consequently, *Pinus nigra* extracts represent promising functional ingredients in cosmetic emulsions, contributing both to skin protection and to the enhancement of oxidative stability during storage [18,19].

pH is a particularly important formulation parameter, as it directly affects ingredient solubility, preservative efficacy, emulsion stability, and skin compatibility. Formulations are typically designed to approximate the physiological pH of the skin (approximately 4.5–5.5) to support the integrity of the acid mantle and preserve barrier function. Deviations from this range may compromise skin homeostasis, potentially leading to irritation, dryness, or inflammatory responses. Consequently, pH optimization and monitoring are fundamental aspects of cosmetic formulation development [4,20]. The prepared emulsions remained physically stable throughout the storage period, maintaining acceptable viscosity profiles despite slight changes over time. Although BP-containing formulations exhibited acidic pH values, no significant pH fluctuations were observed during storage, indicating satisfactory physicochemical stability.

Rheological stability is essential for product spreadability, consumer acceptance, and long-term performance of cosmetic emulsions. Viscosity measurements were conducted at two rotational speeds—50 rpm (Figure 2a) and 100 rpm (Figure 2b), revealing distinct rheological profiles among the formulations over a 28-day storage period. At 50 rpm, all formulations exhibited higher viscosity values compared to measurements at 100 rpm. This rheological profile is particularly desirable for topical formulations, as it facilitates ease of application and spreading while preserving structural stability under static conditions. Cream 1 consistently displayed significantly lower viscosity values across both shear rates, with values ranging from 30 to 40 P at 50 rpm and 17–22 P at 100 rpm. This trend presents both advantages and limitations. From a sensory perspective, reduced viscosity facilitates application and spreading, potentially improving consumer acceptance and tactile properties. Additionally, lower viscosity formulations may require less mechanical force during manufacturing and filling operations. Temporal analysis indicates that most formulations exhibited relatively stable viscosity profiles over the 30-day period, with only minor fluctuations observed. This suggests that the incorporation of active BP extracts does not influence stability.

Color evaluation is commonly performed using standardized colorimetric methods, including the Munsell system and CIELAB color space. In the context of cosmetic formulations, quantitative color assessment is significant, as it provides objective metrics that correlate with consumer perception and product acceptability. In the present study, no significant variations in L* values were observed among the formulations, indicating that the incorporation of extracts did not substantially alter the lightness parameter, with all creams maintaining a white appearance. White or light-colored emulsions are generally preferred in cosmetic applications, as they convey perceptions of purity, cleanliness, and elegance, which are key attributes valued by consumers. Additionally, the neutral color profile ensures versatility across diverse skin tones, as the formulation does not impart visible tint or discoloration upon application [21]. In the CIELAB color space, the positive b* values observed across all emulsions are consistent with a mild yellow tone, with Cream 2 presenting the most prominent yellow deviation, as evidenced by its significantly higher b* value compared to the remaining formulations.

The TPC of different pine tree extracts varies depending on the species and plant part used [22]. Studies have shown that the TPC in pine needle extracts can range from 30 to 150 mg of GAE per gram of dry weight, depending on the extraction method and the specific species of pine [23]. The TPC in pine bark extracts can vary between 100 and 250 mg of GAE per gram of dry weight. Pine bark extracts are also used commercially in products like Pycnogenol, which has been studied for its therapeutic effects, including improving cardiovascular health and reducing oxidative stress [24]. While the TPC of pinecone extracts tends to be lower compared to bark and needles, it can still contribute beneficial antioxidant effects. TPC in pinecone extracts typically ranges from 40 to 120 mg of GAE per gram of dry weight [25]. TPC assessment revealed that Creams 2 and 3 exhibited the highest TPC, with Cream 3 displaying a value of 8.02 ± 0.63 mg GAE/mL, significantly exceeding that of the other formulations. This result suggests that the use of DI as the extraction solvent effectively preserved phenolic compounds, which are known for their antioxidant activity.

Taxifolin is an important bioactive flavonoid present in pine tree extracts, especially from pine needles and bark. Alongside other compounds like proanthocyanidins, flavonoids (quercetin, kaempferol), phenolic acids, and terpenoids, taxifolin contributes significantly to the health benefits associated with pine extracts, like antioxidant and anti-inflammatory properties, which make it a valuable compound for a range of therapeutic and cosmetic applications [26,27]. Although statistical analysis revealed significant differences in taxifolin content among formulations (*p* < 0.05), the absolute values remained relatively similar, ranging narrowly from 1.59 ± 0.01 to 1.84 ± 0.01 mg/1 g cream. From a functional perspective, all formulations retained comparable levels of this bioactive flavonoid, indicating that neither the extraction method nor the solvent employed significantly influenced taxifolin concentration.

The differences observed between the Soxhlet- and reflux-derived formulations should be considered in the context of the chemical composition of the incorporated extracts. Although both procedures employed water and comparable nominal temperature and extraction time, differences in extraction dynamics and thermal history may influence the recovery and stability of individual phytochemicals. In the present study, the reflux-derived formulation (Cream 3) showed a markedly higher TPC than the Soxhlet-derived formulation (Cream 2), whereas the difference in taxifolin content was comparatively small. This indicates that the observed variation in TPC cannot be explained by taxifolin alone and may reflect differences in other phenolic constituents.

Numerous in vitro studies of pine-derived extracts have suggested anti-inflammatory potential, generally associated with the presence of phenolic compounds and other bioactive constituents. These extracts have been reported to reduce selected oxidative stress-related inflammatory mediators and to modulate pathways involved in inflammatory signaling, including NF-κB, depending on the pine species, plant part, extract composition, and experimental model [28,29,30]. However, much of the available mechanistic evidence concerns other pine species, particularly *Pinus pinaster* bark extracts. Therefore, the anti-inflammatory potential of the present black pine (*Pinus nigra*) extract should be interpreted cautiously and requires direct confirmation through dedicated cell-based assays, including measurements of cytokines such as TNF-α and IL-6, as well as relevant inflammatory markers such as COX-2, iNOS, and NF-κB activity.

Tyrosinase inhibition was assessed to determine the creams’ skin-brightening potential. Tyrosinase is a key enzyme in melanin synthesis, and its inhibition helps reduce hyperpigmentation and uneven skin tone [31]. Taxifolin, a prominent flavonoid in pine extracts, has been shown to effectively inhibit tyrosinase activity by binding to the enzyme’s active site, thereby preventing melanin production [32]. This property is particularly relevant in cosmetic formulations aimed at addressing age spots, melasma, and other pigmentation disorders [33]. Ayres et al. concluded that pine bark extract effectively reduces melanin synthesis in vitro by downregulating tyrosinase activity and decreasing pigmentation-related mediators such as endothelin-1 and PPARs [34]. Therefore, BP extracts exhibit significant anti-tyrosinase activity, making them valuable for skin-brightening and depigmentation applications, as well as for treating skin hyperpigmentation induced by various forms of radiation. Moreover, the antioxidant activity of pine extracts complements their anti-tyrosinase effect by mitigating oxidative stress that can exacerbate pigmentation issues.

Creams 2 and 3 demonstrated significantly superior DPPH radical scavenging activity, which was consistent with their higher total phenolic content. This finding supports the established positive correlation between phenolic compound concentration and antioxidant potential, confirming that the bioactive constituents retained their functional activity within the emulsion matrix. Pine tree extracts, particularly those derived from needles, bark, and cones, are rich in phenolic compounds, flavonoids (e.g., taxifolin), and proanthocyanidins, which are well-documented for their strong antioxidant properties [35,36]. These compounds scavenge free radicals, reduce oxidative stress, and prevent lipid peroxidation in biological systems. Studies have demonstrated that extracts from pine bark exhibit high radical scavenging activities in DPPH, ABTS, and ORAC assays [37]. The antioxidant capacity is primarily attributed to the high levels of polyphenols, which neutralize reactive oxygen species (ROS), thereby protecting cellular components like lipids, proteins, and DNA from oxidative damage. This activity has been linked to the potential prevention of age-related diseases, skin aging, and chronic conditions associated with oxidative stress [38,39].

Lower permeability of taxifolin from water-based extracts could be attributed to the hydrophilic nature of the formulation that may limit the interaction with lipophilic membrane layers. Micek et al. suggested that taxifolin, when formulated at a 3% concentration in the Lekobaza^®^ Pharma Cosmetic base cream, effectively penetrates the stratum corneum and enhances skin viscoelasticity without causing irritation [40].

The biological evaluation revealed distinct activity profiles between formulations containing aqueous extracts and those containing oil macerates. However, these differences cannot be attributed solely to the extraction procedure, because the respective aqueous or oil extraction matrices were carried into the final formulations. Water-based extracts (Creams 2 and 3) exhibited the highest antioxidant activity, which corresponded well with their elevated total phenolic content. Exploratory PCA indicated a positive association between TPC and DPPH radical scavenging activity (r = 0.806). These findings are consistent with previous studies indicating that phenolic compounds are the principal contributors to the antioxidant properties of pine extracts [7]. In contrast, formulations containing oil macerates (Creams 4 and 5) showed higher hyaluronidase inhibition and taxifolin permeability. This may result from the higher affinity of lipophilic compounds toward biological membranes as well as the penetration-enhancing properties of olive oil. Interestingly, permeability was negatively correlated with both total phenolic content (r = −0.782) and taxifolin concentration (r = −0.177), suggesting that increasing the concentration of active compounds alone does not necessarily improve their delivery.

Several limitations of the present study should be acknowledged. First, permeability was evaluated using the Skin-PAMPA model, which reflects passive diffusion across an artificial membrane but does not reproduce the full complexity of human skin. Therefore, the observed differences in taxifolin permeability should be regarded as comparative in vitro findings and should be confirmed using ex vivo skin permeation models.

Second, the chemical characterization was limited to TPC and taxifolin determination and therefore does not provide a comprehensive profile of the phytochemical composition of the intermediate extracts. Moreover, the extracts were not standardized to dry extracts prior to formulation. Instead, the aqueous extracts and oil macerates were incorporated together with their respective extraction matrices. Consequently, the present experimental design does not allow the contributions of extraction yield and phytochemical composition to be distinguished from those associated with the extraction medium and its incorporation into the final formulation. The observed differences should therefore be interpreted as formulation-level associations arising from the combined extract preparation and incorporation approach rather than as direct effects of the extraction method alone. Future studies should determine extraction yields, comprehensively characterize the intermediate extracts, remove the extraction solvents, and compare standardized amounts of dry extracts incorporated into an identical formulation matrix.

Third, the reported n values represent replicate measurements performed on the same formulation batch rather than independently prepared formulation batches. Consequently, the inferential statistical results should be interpreted with caution, as technical replication does not account for batch-to-batch variability. Future studies should include multiple independently prepared batches of each formulation to provide a more robust assessment of reproducibility and a stronger basis for inferential statistical comparisons.

Finally, the safety and dermal tolerability of the formulations were not assessed experimentally. Therefore, the present results should be understood as an evaluation of physicochemical and functional properties rather than as evidence of dermal safety. Cytotoxicity, irritation, and sensitization testing will be required before application-related safety claims can be substantiated. Nevertheless, previous studies provide relevant contextual evidence. In a comparative screening of needle extracts from five *Pinus* species, including *P. nigra*, MTT assays showed that normal human MRC-5 fibroblasts were less sensitive than the investigated tumor cell lines [41]. In addition, essential oils obtained from the cones and needles of *P. nigra* have been evaluated topically in in vivo wound-healing and anti-inflammatory models, with no reported adverse effects [42]. These literature data, however, cannot substitute for direct safety assessment of the formulations developed in the present study. Future studies should therefore include cytotoxicity testing using relevant skin cell models, such as keratinocytes and dermal fibroblasts, together with in vitro irritation assessment using reconstructed human epidermis.

From an industrial perspective, these findings provide practical guidance for selecting extraction methods according to the intended cosmetic application. Water extracts may be preferred in formulations designed to maximize antioxidant protection, whereas oil-based extracts appear more suitable for products targeting enhanced dermal delivery and anti-inflammatory effects. Such an approach may facilitate the rational development of multifunctional cosmetic formulations containing *Pinus nigra* extracts.

## 4. Materials and Methods

### 4.1. Materials and Extraction Methods

Needles of Black Pine were obtained commercially from a local supplier tree nursery (Kipos Kalou, Thessaloniki, Greece) and air-dried for 3 days at 25 °C in the dark. The materials were then pulverized into a fine powder using a mechanical grinder and stored in airtight plastic bags until the extraction process. The preparation of extracts from Black Pine was performed using four different methods. Black pine essential oil was purchased from Vessel Essential Oils (Thessaloniki, Greece) and was included as an external commercial comparator representing a commercially available *P. nigra*-derived ingredient. It was not considered equivalent to the laboratory-prepared extracts in terms of raw-material origin or extraction procedure. For the preparation of O/W emulsions, olive oil, shea butter, glycerin, cetearyl alcohol, cetyl alcohol, beeswax, xanthan gum, polysorbate 60 (Tween 60), disodium EDTA, stearic acid, ethylhexylglycerin, and phenoxyethanol were purchased from Novita Group (Thessaloniki, Greece). 2,2-Diphenyl-1-pikrylhydrazyl (DPPH) radical was purchased from Tokyo Chemical Industry (Tokyo, Japan). All other materials and reagents used in this study were of analytical grade purity.

#### 4.1.1. Soxhlet Extraction

For Soxhlet extraction, 15 g of dried and powdered *P. nigra* needles were placed in an extraction thimble, while 200 g of deionized water was placed in the round-bottom flask. The Soxhlet apparatus was heated using a heating mantle set to 70 °C, allowing repeated evaporation, condensation, and cyclic contact of the solvent with the plant material. Extraction was continued for 2 h, starting from the time the heating mantle reached the set temperature. Following extraction, the resulting aqueous extract solution was vacuum-filtered at room temperature to remove insoluble plant material. The filtered aqueous extract was used in liquid form without further concentration or drying prior to incorporation into the emulsion.

#### 4.1.2. Reflux

For the reflux extraction, 15 g of dried and powdered *P. nigra* needles were suspended directly in 200 g of deionized water in a round-bottom flask equipped with a reflux condenser. The aqueous solvent was heated to reflux, with the generated vapor condensing and continuously returning to the extraction flask. No stirring was applied. The heating device was set to 70 °C. Extraction was continued for 2 h, counted from the time at which the heating device reached the set temperature. Following extraction, the resulting aqueous extract solution was vacuum-filtered at room temperature to remove insoluble plant material. The filtered aqueous extract was used in liquid form without further concentration or drying prior to incorporation into the emulsion.

#### 4.1.3. Hot and Cold Maceration Methods

A pool of two equal parts, each of 15 g, was subjected to cold and hot extraction. For cold maceration, 15 g of Black pine powder was extracted with 100 mL of cold olive oil for 48 h. The hot maceration was performed by adding 15 g of the dried fine powder into 100 mL of olive oil at 40 °C, and the water temperature was maintained for 2 h. Extracts were, in each case, subjected to rotary evaporation under reduced pressure at 45 °C to remove trace moisture and volatile water fractions, yielding a completely dehydrated, concentrated Black pine-infused olive oil asset.

### 4.2. Preparation of the O/W Emulsions

The preparation of the O/W emulsions was conducted according to a technique described in previous works from our group [43]. In brief, Table 5 summarizes the two phases and the substances utilized for the preparation of the creams. Regarding the water phase, glycerin and disodium EDTA were dissolved in a beaker at 60 °C, after which xanthan gum was gradually added under continuous stirring to prevent a rapid increase in viscosity. The Soxhlet and reflux aqueous extracts were incorporated into the aqueous phase in their liquid, filtered form without prior drying or concentration, with an equivalent mass of water removed to maintain a constant total formulation weight. The oil phase was heated separately in a water bath at 60 °C until all the substances were dissolved and was then added dropwise to the water phase under constant mechanical stirring (600 rpm) using a 2020 RZR (Heidolph, Schwabach, Germany) mechanical stirrer until complete homogenization. BP essential oil and the two BP extracts were incorporated into the oil phase, with an equivalent mass of olive oil removed to maintain a constant total weight. The prepared emulsions were left under mechanical stirring (400 rpm) for 20 min. Afterwards, a mixture of phenoxyethanol and ethylhexylglycerin (90/10% *w*/*w*) was added as preservatives at room temperature, and mechanical stirring was continued for 10 min. Regarding the O/W formulations, the water phase and the oil phase of the final emulsions were 75% *w*/*w* and 25% *w*/*w*, respectively. One blank emulsion without the addition of Black pine-based compounds was also prepared as the control sample. The final volume/weight ratio of the produced emulsions was 150 mL/200 g. The fabricated formulations were stored in sterilized glass sample jars (250 g) at room temperature.

### 4.3. pH Measurement

The pH stability of the prepared emulsions was studied via pH measurements after 1, 7, 14, 21, and 28 days of storage at room temperature after the preparation of the emulsion. The pH values were read precisely from the instrument by dipping the pH sensor (Microprocessor, WTW, pH 535, Gemini BV, Apeldoorn, The Netherlands), and the mean value of three consecutive measurements (n = 3) was calculated. Variations in the results of pH measurements could not exceed 10% of the initial value for the emulsion to be considered stable.

### 4.4. Viscosity Determination

Viscosity values (n = 3) were determined using the R3 spindle of a Visco Star Plus viscometer (Controla S.A., Thessaloniki, Greece) at 50 and 100 rpm after 1, 7, 14, 21, and 28 days of storage at room temperature after the fabrication of the emulsions. Apparent viscosity determinations were assessed by dipping the R3 spindle of the viscometer for 1 min in each of the studied emulsions, and the obtained values were expressed as Centipoise (cP). Variation in the results of viscosity measurements could not exceed 10% of the initial value for the emulsion to be considered stable.

### 4.5. Color Measurement

The samples were analyzed at room temperature for color variations using a portable MiniScan XE Plus spectrophotometer (HUNTERLAB, Washington, VA, USA). Color evaluation was conducted by measuring changes in the Commission Internationale de l’Éclairage (CIE) (Vienna, Austria) LabCh components. This five-dimensional color space includes L* (lightness), ranging from 0 (black) to 100 (white); a* representing redness (positive values) or greenness (negative values); b* representing yellowness (positive values) or blueness (negative values); C* (chroma, indicating color saturation); and H* (hue angle).

### 4.6. Characterization of Extracts

#### 4.6.1. Total Phenolic Contents (TPC)

The TPC was determined using a previously published method [44]. Prior to analysis, 1 mL of each cream sample was diluted with water, followed by further serial dilutions with water as required to obtain absorbance values within the calibration range. Each vial was filled with 15 µL of Folin–Ciocalteu reagent, 60 µL of 20% sodium carbonate solution, and 25 µL of extract or gallic acid solution. After five minutes of shaking at 600 rpm, the plate was left to stand at room temperature for an additional twenty-five minutes in the dark. The absorbance was then measured at 760 nm using a microplate spectrophotometer (Multiskan GO 1510, Thermo Fisher Scientific, Vantaa, Finland). TPC was calculated from the gallic acid calibration curve, taking into account the corresponding dilution factor, and expressed as mg of gallic acid equivalents (GAE) per mL of cream.

#### 4.6.2. Taxifolin Content

The taxifolin content in the formulations was determined using high-performance liquid chromatography (HPLC). Prior to analysis, the extracts were filtered through a 0.45 μm syringe filter, and 10 μL of each sample was injected into the HPLC system. The stationary phase was a LiChrospher RP-18 column (5 μm, 250 mm × 4 mm) from Merck, Darmstadt, Germany. Formic acid 0.1% (A) and acetonitrile (B) were used in the mobile phase, which had a continuous flow rate of 1.0 mL/min. The gradient flow was as follows: 0–35 min, 2–20% B; 35–55 min, 20–70% B; 55 min, 2% B; 55–60 min, 2% B. The column temperature was maintained at 30 °C. Taxifolin was detected at 290 nm.

#### 4.6.3. Antioxidant Activity

The antiradical activity was assessed using a previously reported DPPH study [1]. A 96-well plate was filled with 175.0 μL of DPPH solution (0.2 mM in methanol) and 25.0 μL of the tested product. The plates were left at room temperature for 30 min while being shaken at 300 rpm in the dark. The absorbance was measured at λ = 517 nm using a plate reader (Multiskan GO 1510, Thermo Fisher Scientific, Vantaa, Finland). All experiments were carried out in nine (n = 9) replicates. The percentage of DPPH scavenging activity (%) was estimated using the following Equation (1):

(1) $$\mathrm{DPPH}\;\mathrm{scavenging}\;\mathrm{activity}\;\left(\%\right)=\;\frac{A_{0}-A_{1}}{A_{0}}\;\times 100\%$$

where *A*_0_ stands for the absorbance of the control (25.0 μL of water mixed with 175.0 of DPPH solution), and *A*_1_ is the absorbance of the sample.

#### 4.6.4. Anti-Inflammatory Activity

The previously published procedure was followed to accomplish the anti-hyaluronidase activity [45]. In short, 10.0 µL of the tested product, 15.0 µL of acetate buffer (pH 4.5, 0.1 mol/L), 25.0 µL of incubation buffer (50 mM, pH 7.0, with 77 mM NaCl and 1 mg/mL of albumin), and 25.0 µL of enzyme solution (30 U/mL) were combined in the well. Following a 15 min shaking incubation period at 37 °C, 25.0 µL of hyaluronic acid (HA) solution (0.3 mg/mL of acetate buffer, pH 4.5) was added, and the mixture was shaken for 45 min at 37 °C. A cetyltrimethylammonium bromide (CTAB) solution in 2% sodium hydroxide (200.0 µL) was then added. A plate reader (Multiskan GO 1510, Thermo Fisher Scientific, Vantaa, Finland) was used to measure the absorbance (λ = 600 nm) following 10 min of incubation at room temperature without shaking. All experiments were carried out in six replicates.

#### 4.6.5. Anti-Tyrosinase Activity

The previously described approach was utilized to test the anti-tyrosinase activity [3]. In short, a 96-well plate was filled with 75.0 µL of phosphate buffer (0.1 M; pH = 6.8), 50.0 µL of 192 U/mL tyrosinase solution, and 25.0 µL of the tested product. At room temperature, the plate was shaken for ten minutes. Next, each well received 50.0 µL of Levodopa (L-DOPA) solution, which was then incubated at 25 °C for 20 min. The control was made using water. The sample’s blank contained the buffer rather than the L-DOPA solution, and the control blank had water in place of the sample and phosphate buffer. After that, the sample’s absorbance at wavelength λ = 475 nm was measured in six (n = 6) replicates.

#### 4.6.6. Penetration of Active Compound

The Skin PAMPA method (skin parallel artificial membrane permeability test) was used to examine the permeability of taxifolin. A two-chamber PAMPA sandwich made up of two 96-well plates makes up the Skin PAMPA model. The lipid-impregnated skin-mimetic membrane is located on the upper plate. 200 μL of the hydration solution (Hydration Solution, Pion Inc., Billerica, MA, USA) was added to each well of pre-coated Skin PAMPA^TM^ sandwich plates (Pion Inc.) to hydrate them overnight before use. Using the previously mentioned HPLC approach in 2.6.2, the quantity of penetrated active chemical after three hours was ascertained in six (n = 6) replicates. The apparent permeability coefficient (P_app_) was calculated via the following Equation (2):

(2) $$P_{\mathrm{app}}=\frac{-\ln\left(1-\frac{C_{A}}{C_{\mathrm{equilibrium}}}\right)}{S\times \left(\frac{1}{V_{D}}+\frac{1}{V_{A}}\right)\times t}$$

where V_D_—donor volume, V_A_—acceptor volume, C_equilibrium_—equilibrium concentration $C_{\mathrm{equilibrium}}=\frac{C_{D}\times V_{D}+\;C_{A}\times V_{A}}{V_{D}+V_{A}}$, C_D_—donor concentration, C_A_—acceptor concentration, S—membrane area, t—incubation time (in seconds).

### 4.7. Statistical Analysis

CIELAB color values were analyzed using Levene’s test for verification of homogeneity of variances, followed by one-way ANOVA with Tukey’s correction for multiple post hoc comparisons. Data on pH and viscosity were analyzed using repeated measures ANOVA, applying the Greenhouse–Geisser correction to adjust for violations of the sphericity assumption. Post hoc pairwise comparisons were adjusted using the Bonferroni correction, while Tukey’s correction was used for multi-group comparisons. For the comparison of total phenolic and taxifolin contents, biological activities, and taxifolin permeability among the formulations, one-way ANOVA followed by Duncan’s multiple range test was applied. The reported n values refer to replicate measurements performed on the same formulation batch. All data are presented as mean ± SD, and *p* < 0.05 was considered statistically significant. All analyses were performed using IBM SPSS Statistics v28.0. Principal component analysis (PCA) was used to analyze correlations using PQStat Software version 1.8.4.142 (2022).

## 5. Conclusions

The present study demonstrated formulation-dependent differences among O/W cosmetic emulsions containing *Pinus nigra*-derived ingredients prepared using different aqueous- and oil-based approaches. Formulations containing aqueous extracts obtained by Soxhlet and reflux extraction showed higher total phenolic content and DPPH radical scavenging activity, whereas formulations containing oil macerates showed higher hyaluronidase inhibition and taxifolin permeability under the experimental conditions employed.

Exploratory PCA indicated a positive association between total phenolic content and DPPH radical scavenging activity, while higher taxifolin permeability and hyaluronidase inhibition were associated with formulations containing oil macerates. These findings should not, however, be interpreted as evidence of an isolated effect of the extraction method or extraction solvent. Because the extracts were not standardized for extraction yield or comprehensive phytochemical composition and were incorporated together with their respective extraction matrices, the contributions of the extraction procedure, extract composition, and extraction medium cannot be independently distinguished in the present experimental design.

The present findings therefore provide a comparative characterization of the investigated formulations rather than evidence of direct causal relationships between extraction conditions and formulation performance. Further studies should include comprehensive phytochemical characterization of the intermediate extracts, determination of extraction yields, and comparison of standardized extracts incorporated into an identical formulation matrix. The use of independently prepared formulation batches and more physiologically relevant permeability models, including ex vivo skin models, would further strengthen the assessment of formulation reproducibility and functional performance.

## Figures and Tables

**Table 1:** Content of active components in each BP emulsion.

Formulation	TPC [mg GAE/1 mL Cream]	Taxifolin Content [mg/1 g Cream]
Cream 1	3.47 ± 0.12 ^c^	1.84 ± 0.01 ^a^
Cream 2	4.15 ± 0.26 ^b^	1.67 ± 0.01 ^c^
Cream 3	8.02 ± 0.63 ^a^	1.79 ± 0.01 ^b^
Cream 4	2.69 ± 0.26 ^d^	1.59 ± 0.01 ^d^
Cream 5	2.32 ± 0.24 ^d^	1.83 ± 0.01 ^a^

Mean values in a column marked with the same letter are not statistically significantly different at *p* = 0.05, according to Duncan’s test.

**Table 2:** Color parameters values obtained from the spectrophotometric assessment of the BP-enriched emulsions.

Emulsion	L* (Lightness)	a* (Red–Green)	b* (Yellow Blue)	C (Chroma)	h° (Hue Angle)	R% (Reflectance)	K/S (Color Strength)
Blank	85.01 ± 2.781	−0.9 ± 0.052	5.76 ± 0.251	5.83 ± 0.21	98.88 ± 5.239	43.19	0.374
Cream 1	83.39 ± 2.56	−0.337 ± 0.015	2.53 ± 0.184	2.55 ± 0.085	97.59 ± 3.175	55.34	0.18
Cream 2	83.89 ± 5.089	−0.084 ± 0.04	7.413 ± 0.499	7.41 ± 0.232	90.65 ± 5.186	44.73	0.34
Cream 3	77.24 ± 3.439	−0.625 ± 0.031	6.26 ± 0.374	6.29 ± 0. 33	95.70 ± 3.213	44.60	0.344
Cream 4	82.67 ± 4.97	−1.093 ± 0.041	5.856 ± 0.310	5.96 ± 0.243	100.57 ± 6.265	52.12	0.22
Cream 5	84.82 ± 3.094	−0.867 ± 0.042	4.913 ± 0. 218	4.99 ± 0.291	100.01 ± 5.147	52.67	0.21

**Table 3:** Antioxidant activity is expressed as the percentage inhibition of the DPPH radical, anti-inflammatory activity as the percentage inhibition of hyaluronidase, and anti-tyrosinase activity as the percentage inhibition of tyrosinase. Values are presented as mean ± standard deviation (n = 3).

Formulation	Antioxidant Activity	Anti-Inflammatory Activity	Anti-Tyrosinase Activity
	Inhibition of DPPH Radical Activity [%]	Inhibition of Hyaluronidase Activity [%]	Tyrosinase Inhibition [%]
Blank	1.01 ± 0.98 ^c^	11.29 ± 1.20 ^e^	9.37 ± 0.81 ^d^
Cream 1	10.48 ± 1.25 ^b^	28.49 ± 1.66 ^d^	51.84 ± 2.49 ^a^
Cream 2	76.78 ± 4.36 ^a^	35.52 ± 3.09 ^c^	42.11 ± 1.30 ^b^
Cream 3	79.72 ± 5.38 ^a^	31.74 ± 2.69 ^d^	28.71 ± 2.45 ^c^
Cream 4	12.19 ± 1.91 ^b^	85.25 ± 0.89 ^a^	28.47 ± 1.33 ^c^
Cream 5	10.24 ± 1.63 ^b^	63.36 ± 1.60 ^b^	26.93 ± 1.90 ^c^

Mean values within a column with the same letter are not significantly different at *p* = 0.05 using Duncan’s test.

**Table 4:** P_app_, apparent permeability coefficient, determined using the Skin-PAMPA model and expressed as cm/s × 10^−6^. Values are presented as mean ± standard deviation (n = 3).

Formulation	P_app_ × 10^−6^ [cm/s]
Cream 1	38.58 ± 2.11 ^b^
Cream 2	31.14 ± 3.89 ^c^
Cream 3	26.99 ± 2.00 ^c^
Cream 4	50.56 ± 2.82 ^a^
Cream 5	45.65 ± 2.20 ^a^

Mean values within a column with the same letter are not significantly different at *p* = 0.05 using Duncan’s test.

**Table 5:** Formulations and ingredients used for the aqueous and oil phases for the prepared emulsions. Quantities are given for 200 g cream. The water phase and the oil phase constituted 75% and 25% *w*/*w* of the final emulsion, respectively. In Creams 2 and 3, the aqueous extract replaced an equivalent mass of water; in Creams 1, 4, and 5, the essential oil or oil macerate replaced an equivalent mass of olive oil. The “-” symbol indicates that the ingredient was not included in that formulation.

Ingredients	*w*/*w*	Blank	Cream 1	Cream 2	Cream 3	Cream 4	Cream 5
Water phase							
Water	70%	140 g	140 g	118 g	118 g	140 g	140 g
Glycerin	3.5%	7 g	7 g	7 g	7 g	7 g	7 g
Disodium EDTA	0.5%	1 g	1 g	1 g	1 g	1 g	1 g
Xanthan gum	1%	2 g	2 g	2 g	2 g	2 g	2 g
Active ingredient							
BP essential oil	11%	-	22 g	-	-	-	-
Soxhlet water extract	11%	-	-	22 g	-	-	-
Reflux water extract	11%	-	-	-	22 g	-	-
Hot oil BP extract	11%	-	-	-	-	22 g	-
Cold oil BP extract	11%	-	-	-	-	-	22 g
Oil phase							
Olive oil	11%	22 g	-	22 g	22 g	-	-
Tween 60	2%	4 g	4 g	4 g	4 g	4 g	4 g
Cetearyl alcohol	2%	4 g	4 g	4 g	4 g	4 g	4 g
Cetyl alcohol	2%	4 g	4 g	4 g	4 g	4 g	4 g
Stearic acid	2%	4 g	4 g	4 g	4 g	4 g	4 g
Beeswax	2%	4 g	4 g	4 g	4 g	4 g	4 g
Shea Butter	2%	4 g	4 g	4 g	4 g	4 g	4 g
Preservatives							
Ethylhexylglycerin	1%	2 g	2 g	2 g	2 g	2 g	2 g
Phenoxyethanol	1%	2 g	2 g	2 g	2 g	2 g	2 g

## Data Availability

The original contributions presented in this study are included in the article. Further inquiries can be directed to the corresponding author.

## Abbreviations

The following abbreviations are used in this manuscript:

BP	Black pine
O/W	Oil-in-water
TPC	Total phenolic content
PCA	Principal Component Analysis
